# Novel Synergistic Probiotic Intervention: Transcriptomic and Metabolomic Analysis Reveals Ameliorative Effects on Immunity, Gut Barrier, and Metabolism of Mice during *Salmonella typhimurium* Infection

**DOI:** 10.3390/genes15040435

**Published:** 2024-03-29

**Authors:** Muhammad Junaid, Hongyu Lu, Yixiang Li, Yu Liu, Ahmad Ud Din, Zhongquan Qi, Yi Xiong, Jianhua Yan

**Affiliations:** 1 Medical College, Guangxi University, Nanning 530004, China; drjunaid42@st.gxu.edu.cn (M.J.); hongyulu2000@163.com (H.L.); lyixiang@foxmail.com (Y.L.); liuyu@gxu.edu.cn (Y.L.); yxyyz@gxu.edu.cn (Z.Q.); 2 Plants for Human Health Institute, North Carolina State University, 600 Laureate Way, Kannapolis, NC 28081, USA; 3 Guangxi Center for Animals Disease Control and Prevention, Nanning 530004, China

**Keywords:** synergistic probiotics, immunity, metabolism, gut barrier, *Lactobacillus acidophilus*, Lactobacillus plantarum, *Salmonella typhimurium*

## Abstract

*Salmonella typhimurium* (*S. typhimurium),* a prevalent cause of foodborne infection, induces significant changes in the host transcriptome and metabolome. The lack of therapeutics with minimal or no side effects prompts the scientific community to explore alternative therapies. This study investigates the therapeutic potential of a probiotic mixture comprising *Lactobacillus acidophilus* (*L. acidophilus 1.3251*) and *Lactobacillus plantarum* (*L. plantarum 9513*) against *S. typhimurium*, utilizing transcriptome and metabolomic analyses, a novel approach that has not been previously documented. Twenty-four SPF-BALB/c mice were divided into four groups: control negative group (CNG); positive control group (CPG); probiotic-supplemented non-challenged group (LAPG); and probiotic-supplemented *Salmonella*-challenged group (LAPST). An RNA-sequencing analysis of small intestinal (ileum) tissue revealed 2907 upregulated and 394 downregulated DEGs in the LAPST vs. CPG group. A functional analysis of DEGs highlighted their significantly altered gene ontology (GO) terms related to metabolism, gut integrity, cellular development, and immunity (*p ≤* 0.05). The KEGG analysis showed that differentially expressed genes (DEGs) in the LAPST group were primarily involved in pathways related to gut integrity, immunity, and metabolism, such as MAPK, PI3K-Akt, AMPK, the tryptophan metabolism, the glycine, serine, and threonine metabolism, ECM–receptor interaction, and others. Additionally, the fecal metabolic analysis identified 1215 upregulated and 305 downregulated metabolites in the LAPST vs. CPG group, implying their involvement in KEGG pathways including bile secretion, propanoate metabolism, arginine and proline metabolism, amino acid biosynthesis, and protein digestion and absorption, which are vital for maintaining barrier integrity, immunity, and metabolism. In conclusion, these findings suggest that the administration of a probiotic mixture improves immunity, maintains gut homeostasis and barrier integrity, and enhances metabolism in *Salmonella* infection.

## 1. Introduction

*Salmonella* species are notable Gram-negative organisms that affect humans, animals, and poultry, which are capable of causing a wide range of diseases, ranging from mild foodborne diarrhea to severe systemic issues, and remain a serious significant global public health concern [1,2]. It is reported that more than 350,000 people die worldwide from *Salmonella* infection each year [3]. Among *Salmonella* strains, *S. typhimurium* is accountable for most human salmonellosis cases worldwide [4,5,6]. The emergence of multidrug-resistant strains of *S. typhimurium* has led to an alarming rise in the incidence of *Salmonella* infections [4], encouraged by the indiscriminate use of antibiotics, which has reduced the effectiveness of conventional antibiotics therapy due to antibiotic resistance [7]. Additionally, *S. typhimurium,* characterized as an intracellular pathogen capable of infecting and replicating within both phagocytic and non-phagocytic cells, further undermines antibiotic effectiveness [8]. Consequently, finding alternative therapies is imperative, and probiotics offer a promising avenue. Probiotic bacteria have been extensively studied for their ability to reduce the severity of intestinal and associated diseases [9,10,11]. Probiotics are live microorganisms that confer health benefits to their host when taken in appropriate amounts [12]. They have been shown to have significant impacts on the gastrointestinal tract, such as boosting metabolic function [13], combating infection [14], modulating immunological function, and alleviating symptoms of gastro-intestinal (GI) disease [15,16]. Different probiotic strains have specific functions, while the benefits of combining numerous strains with a diverse range of beneficial bacteria, with each contributing a unique mechanism of action, are being highlighted in emerging research [17,18,19]. Of significant note is a probiotic mixture containing *L. acidophilus* and *L. plantarum*, calling attention to its potential [20,21]. After a thorough evaluation of their probiotic qualities, *L. plantarum* and *L. acidophilus* were shown to be effective in reducing sepsis, enhancing host growth, managing inflammation associated with enteritis, eliminating harmful bacteria, and collectively improving intestinal health [22,23,24,25,26].

After passing through the intestinal tract, probiotics face the challenge of establishing themselves in ecological niches with native microbes and enduring harsh conditions to ensure survival [27]. In general, probiotics can potentially engage intestinal cells directly, maintaining epithelium while interacting with pathogenic microorganisms in the gut, thereby aiding and preventing diseases [28,29,30]. Thus, grasping the fundamental mechanisms underlying these intricate probiotic interactions within the gastrointestinal system is paramount. Transcriptome and metabolome analyses are vital in unveiling a comprehensive view of host alterations in response to probiotics [31]. Transcriptome analysis has been exploited to investigate cellular reactions to probiotics in vivo, providing valuable insights into molecular shifts [32,33]. Simultaneously, metabolomics shed light on how probiotics impact intestinal microbial metabolites, producing advantageous substances that fortify the intestinal barrier and improve gut immunity and metabolism, thereby supporting host health [34]. Additionally, probiotics can have a range of beneficial metabolites, including fatty acids, polysaccharides, and short-chain fatty acids, which can translocate across the intestinal epithelium and enter the systemic circulation [35]. Previous studies highlighting the robust effects of *L. acidophilus* and *L. plantarum* strains from diverse perspectives [36,37,38] convinced us to investigate these specific strains in the *Salmonella*-induced mouse model.

The purpose of this study is to evaluate the effectiveness of a probiotic mix containing *L. acidophilus* (CGMCC 1.3251) and *L. plantarum* (CGMCC 9513) as an alternative treatment for *S. typhimurium* infection. It also looks into global transcriptional changes and fecal metabolite modulation to gain nuanced insights into probiotic-host–pathogen interactions and help develop efficacious alternative therapies against *S. typhimurium* infections.

## 2. Materials and Methods

In this study, a probiotic mixture of *L. acidophilus* (CGMCC 1.3251) and *L. plantarum* (CGMCC 9513) was used, sourced from Tianjin Shengji Group Co., Ltd., Tianjin, China. The bacterial suspension was prepared by combining equal proportions (1:1) of the two *Lactobacillus* strains, resulting in a final concentration of 6 × 10^5^ cfu/mL of lactic acid bacteria. *S. typhimurium* was procured from the National Center for Medical Culture Collection China, cultured on selective mediums of bismuth sulfite agar and Selenite cystine broth, and incubated at 37 °C for 24 h. The resulting colonies of *S. typhimurium* were harvested and adjusted to a final concentration of 2 × 10^6^ cfu/mL. All experiments and animal handling procedures adhered to the guidelines and regulations of the Ethical Committee of Guangxi University (GXU-2023-0125), ensuring that ethical standards were maintained throughout the study.

### 2.1. Animals, Experimental Design, and Treatment

In this study, we obtained a cohort batch of specifically pathogen-free (SPF) female BALB/c mice, aged between 4 and 5 weeks, from Bay Fu Biotechnology Co., Limited, Beijing, China. Upon their arrival, the mice underwent a 7-day acclimation period to adapt to the laboratory environment. During the experimental and acclimation periods, these mice were provided free access to food and water. They were housed in a controlled environment with a 12 h light/dark cycle at a temperature of 22 ± 2 °C and relative humidity of 44 ± 0.2% to ensure optimal conditions for their well-being and optimize the reliability of the experimental outcomes.

The experiment spanned three weeks. Twenty-four mice were divided into four groups (n = 6): (1) control negative group (CNG), a non-challenged, non-treated group, containing mice that received a regular diet only; (2) control positive group (CPG), *Salmonella*-challenged, non-treated mice, receiving a standard diet and infected with *S. typhimurium* on the 7th day; (3) *L. acidophilus* + *L. plantarum*-mixture-fed, non-challenged group (LAPG), containing mice receiving a probiotics mixture without *Salmonella* infection; and (4) *L. acidophilus* + *L. plantarum*-mixture-fed, *Salmonella*-challenged group (LAPST), containing mice fed with a probiotic mixture and infected with *S. typhimurium*. On day seven, the CPG and LAPST groups were intragastrically infected with 0.2 mL of *S. typhimurium* suspension containing 2 × 10^6^ cfu/mL via a feeding needle. In contrast, mice in the CNG and LAPG groups were treated via the same method to stimulate similar handling stress. After infection, the LAPG and LAPST groups were treated daily with 6 × 10^5^ cfu/mL of probiotic mixture for two weeks using the same method. To prevent cross-contamination, the *Salmonella*-infected and uninfected groups were housed separately. Fourteen days post-infection, three mice per group were euthanized by cervical dislocation for further analysis.

### 2.2. Sample Collection and Transcriptome Data Analysis

Three mice per group were euthanized by cervical dislocation on the 14th day post-infection, and a 2 cm intestinal (ileum) tissue sample was collected. The TRIzol method [39] was used to isolate total RNA following the manufacturer’s instructions (Magigene Biotechnology, Guangzhou, China). RNA’s integrity, purity, and concentration were then determined by Nano-Drop One (Thermo Fisher Scientific, Waltham, MA, USA) and the Aligent 4200 system (Aligent Technologies GmbH, Waldbronn, Germany).

#### 2.2.1. cDNA Library Construction of Transcriptome Samples

The QuantSeq 3′-mRNA-Seq Library Prep Kit for Illumina, supplied by Lexogen, was employed to create QuantSeq libraries using 120 ng of RNA. During reverse transcription, RT primers such as oligo-dT with 5′ adapters compatible with Illumina captured the 3′ end of the mRNA molecules. Following the hydrolysis of RNA strands using an RNase H-specific hydrolase, a second DNA–RNA hybrid was created using arbitrary primers. Carboxyl-modified GE Sera-Mag Magnetic Speed beads were utilized to isolate cDNA fragments between (150 and 200 bp). Illumina index primers were used in conjunction with 2× PfuMax HiFi PCR ProMix and VAHTS Multiplex Oligos Set 4 for the PCR amplification. Carboxylated GE Sera-Mag Magnetic Speed beads were used to purify the PCR products, and the insert size was measured by the Qsep400. To cluster the index-coded samples, cBot cluster formation technology was followed by sequencing on the Illumina Novaseq 6000 platform, generating 150 base pair (bp) paired-end reads per sample.

#### 2.2.2. Raw Data Processing and Screening of Differentially Expressed Genes

Low-quality reads were filtered and the fastp (version 0.23.2) software was used to assess the remaining clean reads’ Q20, Q30, and GC contents, ensuring high-quality data acquisition [39]. After that, the clean data were mapped to the Mus musculus reference genomes obtained from ncbi.com. Differential gene expression between groups was analyzed by using DESeq2 (v1.34.0) software [40]. The criteria for identifying differentially expressed genes (DEGs) were set as FDR ≤ 1, log2FC ≥ 1, and *p* value ≤ 0.05.

#### 2.2.3. Analysis of Gene Ontology (GO) and Kyoto Encyclopedia of Genes and Genomes (KEGG) Pathways in Genes Exhibiting Differential Expression

At this stage, the genes that exhibited notable differences in expression were analyzed for the enrichment of specific biological processes and pathways using GO terms and KEGG pathways. To explore GO and KEGG pathways, the R package (v4.2.2) [41] and KOBAS (3.0) [42] were used.

### 2.3. DEGs’ Validation by qRT-PCR

To validate the RNA-seq data obtained from comparing the LAPST and CPG groups, a subset of five differentially expressed genes (DEGs) was selected for further verification. The primers used for validation are listed in Table S1. The β-actin gene was chosen as an internal reference gene for normalization. To assess the consistency of the RNA-seq results, each sample was replicated three times, and the transcriptional levels of each gene were calculated using the 2^−∆∆Ct^ method [43].

### 2.4. Metabolome Data Processing and Analysis

The intestinal fecal contents sample (100 mg) was combined with a 400 µL extract solution composed of cold methanol: acetonitrile: water in a 2:2:1 *v*/*v* ratio, and a stable isotope internal standard.

The mixture was vortexed for at least 30 s, ground for 4 min, and sonicated at 35 Hz for 5 min. Following this, it was incubated for 1 h at −40 °C and then centrifuged at 12,000 rpm for 15 min at 4 °C. The resultant was collected, filtered, and then subjected to detection in a 2 mL injection vial [44,45]. All samples were pooled into quality control (QC) samples, ensuring an equal amount of supernatant.

#### 2.4.1. LC-MS Detection of Differential Metabolites and KEGG Pathways’ Analysis

An Orbitrap MS Q Exactive HFX mass spectrometer (Thermo Fisher Scientific) was connected to a 2.1 mm × 100 mm, 1.7 μm UPLC BEH amide column in a UHPLC system (Vanquish, Thermo Fisher Scientific) for the LC-MS study. The mobile phase consisted of water (pH 9.75) and acetonitrile (B), and the samples containing 25 mmol/L ammonium acetate and 25 mmol/L ammonia hydroxide were used. The auto-sampler temperature was maintained at 4 °C and the injection volume was 3 µL. Thermo’s Xcalibur acquisition (2.1.0) software guided the QE HFX mass spectrometer by acquiring MS/MS spectra in information-dependent acquisition (IDA) mode. In the NCE mode, the collision energy was adjusted to 10/30/6, and the total complete MS resolution was set at 60,000.

#### 2.4.2. Orthogonal Projection to Latent Structures Discriminant Analysis (OPLS-DA) and Principal Component Analysis

We employed the OPLS-DA model to identify group separation and find noticeably changed metabolite levels. Differentially expressed metabolites (DEMs) were analyzed using the following criteria: variable importance in projection (VIP) value ≥ 1, *p* value ≤ 0.05, and log2FC > 1. Subsequently, the biological significance of the metabolites was determined by performing a functional analysis of metabolic pathways utilizing the KEGG database [46].

## 3. Results

### 3.1. Transcriptomic Data Analysis

To delve into the possible molecular mechanisms of the probiotic mixture (*L. acidophilus* + *L. plantarum*) in preventing *Salmonella* infection in mice, we conducted an RNA sequencing analysis on small intestinal (ileum) tissue samples from four groups (control negative group (CNG), *Salmonella*-challenged untreated group (CPG), non-challenged probiotic-fed group (LAPG), and *Salmonella*-challenged probiotic-treated group (LAPST)). We performed an RNA sequencing of 12 small intestinal (ileum) libraries, each consisting of three replicate samples, which yielded 119,012,040 150 bp pair-end raw reads with a cumulative length of 17.84 Gb. After trimming and filtering operations to remove low-quality reads and adapters, we obtained a total of 109,279,661 high-quality reads with an average length of 15.77 gigabases (Gb) and an average quality score of 98.325%, as measured by the Phred score. The average GC content across all samples was measured at 43.39%, reflecting a consistent GC content profile (Table S2). These findings reveal abundant RNA sequencing data and a relatively stable GC content across the analyzed samples.

#### 3.1.1. Differential Regulation of Genes Induced by Exposure to *S. typhimurium* and Probiotics

**Figure 1:**
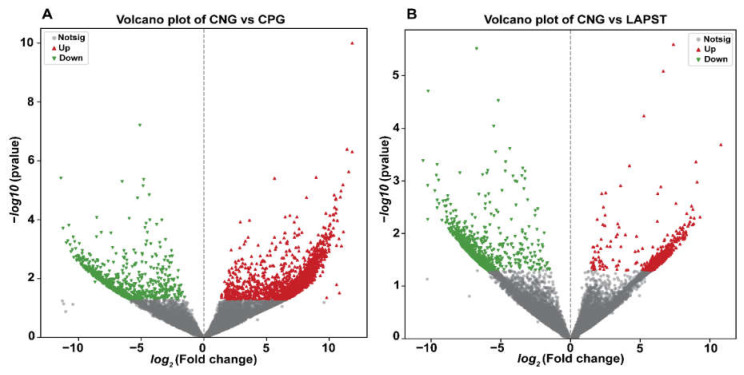

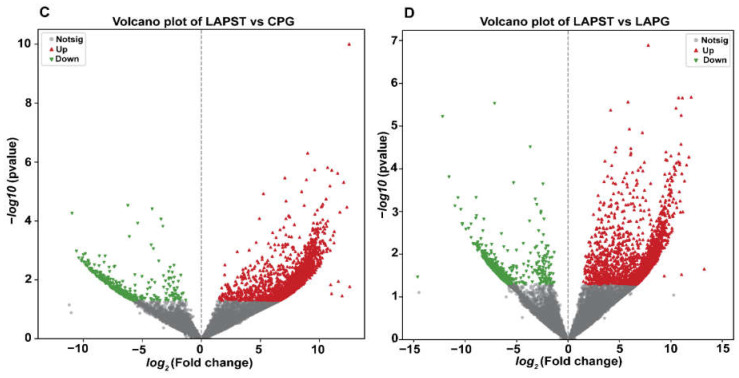
Volcano plots showing differentially up- and downregulated genes between groups. (**A**) CNG vs. CPG; (**B**) CNG vs. LAPST; (**C**) LAPST vs. CPG; (**D**) LAPST vs. LAPG. Red dots show upregulated, green dots show downregulated, and gray dots shows normally expressed genes.

A total of 2335 upregulated and 654 downregulated DEGs were identified in the CNG vs. CPG group comparison. In the CNG vs. LAPST comparison, 488 upregulated and 886 downregulated DEGs were observed. Additionally, 2907 upregulated and 394 downregulated DEGs were detected in the LAPST vs. CPG comparison, whereas 2434 upregulated and 543 downregulated DEGs were found in the LAPST vs. LAPG group comparison (Figure 1A–D and Table S3).

#### 3.1.2. Genes Regulated by Probiotic Treatment Involved in Immunity, Gut Barrier Integrity, and Metabolism

Several genes related to immunity, gut barrier integrity, and metabolism showed significant upregulation in the *Salmonella*-infected probiotics-treated group (LAPST) compared to the *Salmonella*-infected non-treated group (CPG). The upregulated genes involved in immunity mainly include TNF receptor-associated protein 1 (Trap1), CD9 antigen (Cd9), interleukin 7 (Il7), TNF receptor-associated factor 3 (Traf3), chemokine C-C motif ligand 8 (Ccl8), TNF receptor-associated factor 6 (Traf6), chemokine (C-C motif) ligand 22 (Ccl22), and others (Table S4). Similarly, genes involved in gut barrier integrity such as superoxide dismutase 1 (SOD-1), heat shock protein 2,5,6,9,14 (Hspb2,5,6,9,14), glutathione S-transferase, alpha 4 (Gsta4), chloride channel accessory 4A (Clca4a), and integrin alpha L (Itgal) were also upregulated (Table S4). In addition, genes involved in metabolism, including Acsl4, Adcy3, Cox18, Agpat4, Cyp2s1, Cox8a, Uck2, and others, were significantly upregulated in the probiotics-treated group infected with *Salmonella* compared to the non-treated group (Table S4).

#### 3.1.3. Gene Ontology Annotation and Enrichment Analysis

**Figure 2:**
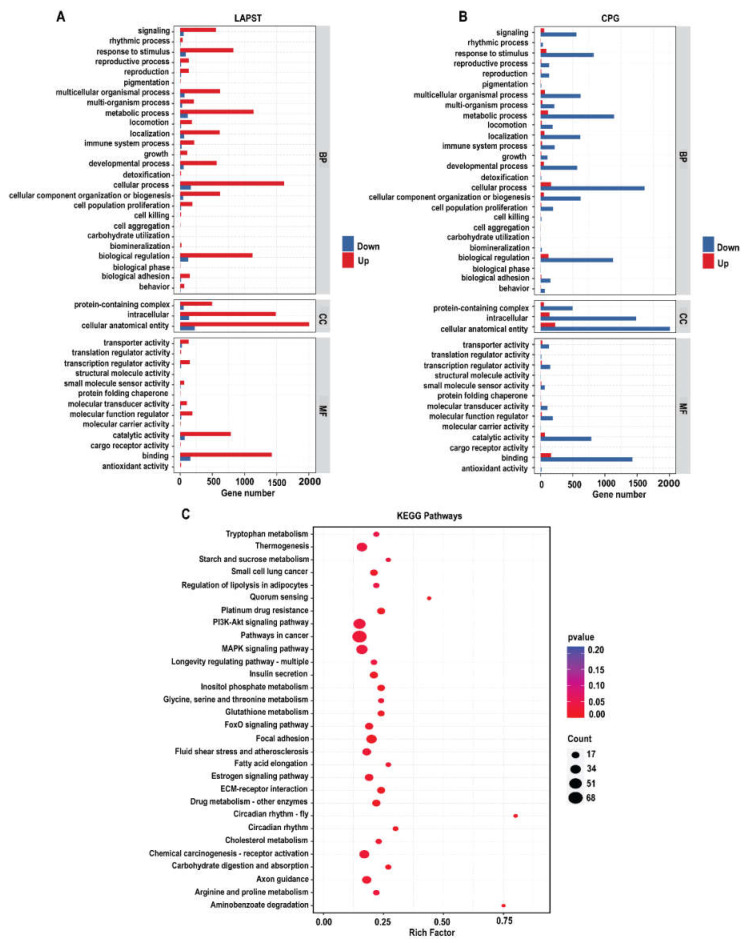
GO and KEGG enrichment analysis in LAPST vs. CPG group: (**A**) up- and downregulated GO terms in LAPST; (**B**) up- and downregulated GO terms in CPG; (**C**) top 30 significantly enriched KEGG pathways (*p* ≤ 0.05).

To investigate the molecular mechanisms underlying probiotic supplementation at the genetic level, we analyzed 3301 differentially expressed unique genes between LAPST and CPG groups. These DEGS were classified into functional groupings using the gene ontology (GO) classification system, which includes biological processes (BP), cellular components (CC), and molecular functions (MF). A total of 1843, 2022, and 1871 GO terms were significantly enriched (*p ≤* 0.05) for upregulated DEGs, while 211,227 and 212 GO terms were enriched for downregulated DEGs in the biological process (BP), cellular component (CC), and molecular function (MF) in LAPST compared to CPG (Figure 2A,B and Table S5). The enriched GO terms in BP included cellular processes, metabolic processes, growth, immune system processes, developmental processes, reproduction, and biological regulation. The terms enhanced in CC included proteins containing complex, intracellular, and cellular anatomical entities. The enriched GO terms in MF exhibited catalytic, antioxidant, binding, molecular function regulator, transcription, and translation regulator activities (Figure 2A,B). The annotated Gene Ontology (GO-BP), (GO-CC), and (GO-MF) terms underwent systematic classification into more comprehensive categories, as shown in (Figure S1A–C and Table S6).

#### 3.1.4. Kyoto Encyclopedia of Genes and Genomes (KEGG) Pathways Analysis

To explore the regulatory pathways affected by probiotic feeding, we mapped the 3301 DEGs to the Kyoto Encyclopedia of Genes and Genomes (KEGG) database for pathways enrichment analysis. Several pathways related to immunity, metabolism, and gut barrier integrity were found to be affected. Upregulated pathways related to immunity included circadian rhythm, extra-cellular matrix (ECM) receptor interaction, the mitogen-activated protein kinase (MAPK) signaling pathway, PI3K-Akt signaling pathway, the insulin resistance Forkhead box o (FOXO) signaling pathway, focal adhesion, AMP-activated protein kinase (AMPK) signaling pathways, and leukocyte trans-endothelial migration. Similarly, the pathways related to metabolism, including inositol phosphate metabolism, tryptophan metabolism, drug metabolism, glycine, serine, and threonine metabolism, starch and sucrose metabolism, arginine and proline metabolism, insulin secretion, and steroid biosynthesis pathways (*p* ≤ 0.05), were upregulated due to probiotic supplementation exposure (Figure 2B and Table S7).

### 3.2. Validation of DEGs’ by qRT-PCR

To ensure the reliability of our RNA-seq results, we validated three upregulated genes (SOD-1, IL-10, and Cldn-1) and two downregulated genes (Cox-3 and Tnfsf14) using qRT-PCR. Our results showed a strong correlation between qRT-PCR and RNA-seq fold change values, as depicted in Figure S2. The alignment between the qRT-PCR and RNA-seq results demonstrates the reliability of transcriptome analysis.

### 3.3. Effects of Probiotic Treatment on Gut Metabolites (LC/MS Analysis)

A metabolomic analysis was performed on fecal intestinal contents from the probiotic-treated LAPST group challenged with *Salmonella* compared to the CPG group (*Salmonella*-challenged non-treated group) using non-targeted liquid chromatography-mass spectrometry (LC-MS). Data quality control was carried out following the qualitative and quantitative metabolites analysis to guarantee the precision and dependability of the results. Multivariate statistical analyses were carried out for metabolites, including principal component analysis (PCA) and orthogonal partial least squares discriminant analysis (OPLS-DA). The PCA analysis revealed distinct sample separation, with PC1 accounting for 41.35% and PC2 for 26.06% of the variance. Furthermore, the OPLS-DA model showed significant differences, with R2X (cum) = 0.541, R2Y (cum) = 0.994, and Q2 (cum) = 0.88 (Figure S3A–D). The closer the R2 values are to 1, the more reliable the model, which indicates a clear classification between the infected and treated groups.

#### 3.3.1. Differential Metabolites Analysis

**Figure 3:**
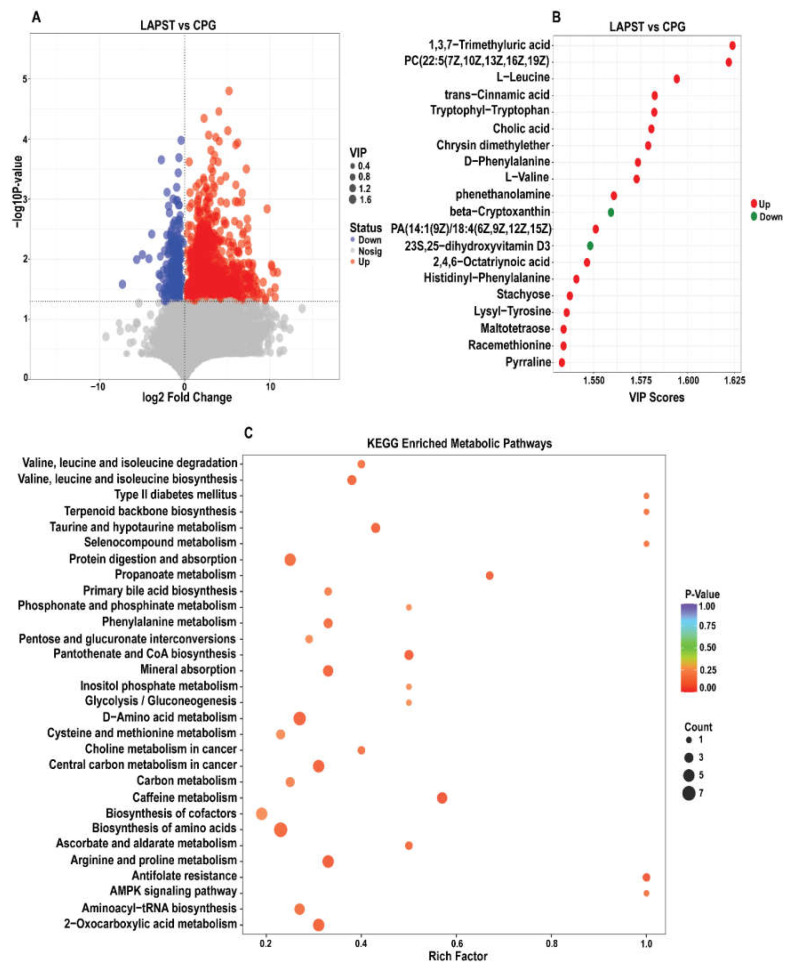
(**A**) Volcano plot showed significantly expressed up- and downregulated metabolites between LAPST vs. CPG. (**B**) The top 20 metabolites exhibit significant variable importance in projection (VIP ≥ 1) score in the LAPST vs. CPG group. (**C**) Top 30 significantly enriched KEGG metabolic pathways between LAPST vs. CPG. (*p ≤* 0.05).

A total of 1520 significantly altered metabolites were identified as meeting the criteria of *p*-value ≤ 0.05, VIP ≥ 1, and log2FC│ > 1, with 1215 upregulated and 305 downregulated metabolites in the LAPST versus CPG group (Figure 3A and Table S8). The top 20 upregulated and downregulated metabolites, as shown in Figure 3B, include L-leucine, 1,3,7-trimethyluric acid, tryptophyl−tryptophan, trans-cinnamic acid, cholic acid, 2-hydroxybutyric acid, D-phenylalanine, L-valine, lysl-tyrosine, 2,4,6-octatriyonic acid, N-acetyl-L-leucine, taurine, phenethanolamine and cholic acid. β-cryptoxanthin and 23S, 25−dihydroxy vitamin D3 were downregulated by the probiotic’s treatment compared to the *Salmonella*-infected non-treated group. Moreover, a comprehensive correlation analysis of the top 100 differential metabolites was conducted using the VIP score, as depicted in Figure S4. This investigation corroborates the interconnectedness of the samples and metabolites within each group, providing valuable insights into the metabolic changes occurring across the different samples.

#### 3.3.2. Differential Metabolites KEGG Metabolic Pathways Analysis

To explore the impact of probiotics treatment on *S. typhimurium*, the differential metabolites were mapped to the KEGG pathways with (*p* ≤ 0.05) as the assessment criteria. The top 30 enriched KEGG metabolic pathways (Figure 3C), comprised caffeine metabolism, arginine and proline metabolism, pantothenate and CoA biosynthesis, bile secretion, 2-oxocarboxylic acid metabolism, D-amino acid metabolism, propanoate metabolism, aminoacyl-tRNA biosynthesis, primary bile acid biosynthesis, amino acid biosynthesis, protein digestion and absorption, valine, leucine and isoleucine biosynthesis, taurine and hypo-taurine metabolism and phenylalanine metabolism. These pathways were significantly enriched in the probiotic-treated LAPST group compared to the *Salmonella* infected non-treated CPG group.

## 4. Discussion

Infection with *S. typhimurium* can lead to enteric inflammation in the host, characterized by acute intestinal inflammation and diarrhea. Notably, research has highlighted that *S. typhimurium* is a primary cause of foodborne illnesses globally, underscoring the significant public health implications of this pathogen [47]. *Salmonella* infection is well documented to disrupt the digestive process, hinder nutrient absorption, disrupt the delicate balance of gut physiology and metabolism, and suppress the host’s immune system [48,49,50]. Antibiotics are commonly administered for treating *Salmonella* infections, but the rise of antibiotic resistance [51] has prompted a search for alternative methods. Probiotics have emerged as a promising alternative [52,53,54]. Numerous research studies have demonstrated that probiotic microorganisms can improve the digestion of nutrients, maintain the balance of gut bacteria, strengthen the gut barrier integrity, fend off harmful organisms, and modulate both humoral and cellular immune responses [55]. Through various modes of action, including transcriptome and metabolome analyses, we investigated the potential beneficial effects of a probiotic mixture in counteracting the adverse effects of *Salmonella* infection. Our findings revealed that several genes encoding proteins involved in immunity, gut barrier integrity, homeostasis, and metabolism were upregulated in the probiotic-treated group (LAPST) compared to the non-treated group (CPG) infected with *Salmonella*. These current findings corroborate previous studies that demonstrated that probiotic supplementation modulates differentially expressed genes (DEGs) linked to immunity, homeostasis, and metabolism in the gut tissue of mice [56,57], piglets [58], and chickens [59,60]. Positive changes in genes related to immunity and metabolism, and pathways that improve the host’s physiological state and general function, are known to be modulated by beneficial microorganisms [61]. Furthermore, early exposure to probiotic microbes profoundly impacts the development of immunological components and has a long-lasting impact on the host’s physiology [62].

**Figure 4:**
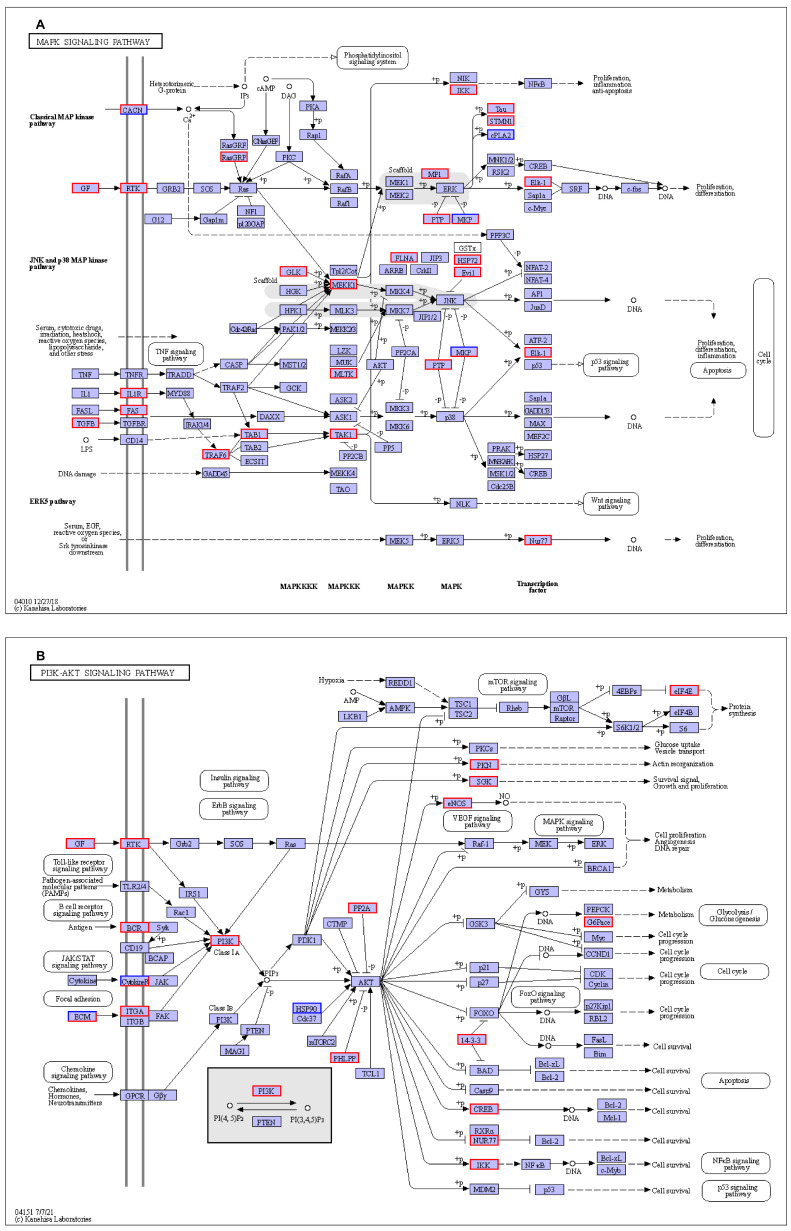
Significantly altered KEGG pathways in LAPST versus CPG group comparison. (**A**) MAPK signaling pathway DEGs expression patterns. (**B**) PI3K/Akt signaling pathway DEGs expression patterns. Red-highlighted genes indicate patterns of upregulated expression.

To further explore the roles of differentially expressed genes (DEGs) and their relationships with biological pathways, we used Gene Ontology (GO) and Kyoto Encyclopedia of Genes and Genomes (KEGG) analyses. Our findings demonstrated the significant enrichment of numerous GO keywords and KEGG pathways linked with gut integrity, immunity, and metabolism in the LAPST group, which received probiotic treatment following the *Salmonella* challenge, compared to the untreated control group (CPG). The key enriched KEGG pathways included ECM–receptor interaction, MAPK, PI3K-Akt, Foxo, and AMPK signaling pathways, inositol phosphate metabolism, tryptophan metabolism, glycine, serine, and threonine metabolism, focal adhesion, arginine, and proline metabolism, and steroid biosynthesis pathways (Figure 2B and Table S7). The immune system plays a crucial role in protecting the host against infections. During infection, immune cells engage several signaling pathways that provide defense mechanisms against pathogens [63]. MAPK signaling pathways play a significant role in the first line of defense against infections, coordinating immune cell regulation, proliferation, and survival, and also influencing inflammatory cytokines [64]. Yun-Gi Kim et al. [65] discovered, in a parallel investigation, that supplementation with probiotic *Lactobacillus casei* greatly elevated the MAPK signaling pathway, which is consistent with our findings. Similarly, the PI3K/Akt signal pathway exhibited a notable inhibitory effect on interleukin-8 (IL-8) responses induced by *Salmonella* infection when *Lactobacillus rhamnosus GG* was administered prior to infection [66]. This result is consistent with our finding, which revealed that the probiotic-administered group exhibited a notable enhancement in the PI3K-Akt signaling pathway (Figure 2B). Yanping Wu et al.’s study [67] demonstrated that *L. plantarum* induced AMPK-mediated autophagy to inhibit NLRP3 inflammasome and *Salmonella* infection in IPEC-J2 cells, consistent with our results. Furthermore, FOXO signaling pathways regulate cell growth, differentiation, death, and immunity [68]. In our experiment, 40, 47, 19, and 19 genes were differentially expressed (DEGs) in response to probiotic treatment, with enrichment in signaling pathways including MAPK, PI3K/Akt, AMPK, and FoxO (Figure 4A,B and Figure S5A,B). These results imply that the administration of the probiotic mixture triggers the protective mechanism against *Salmonella* infection.

**Figure 5:**
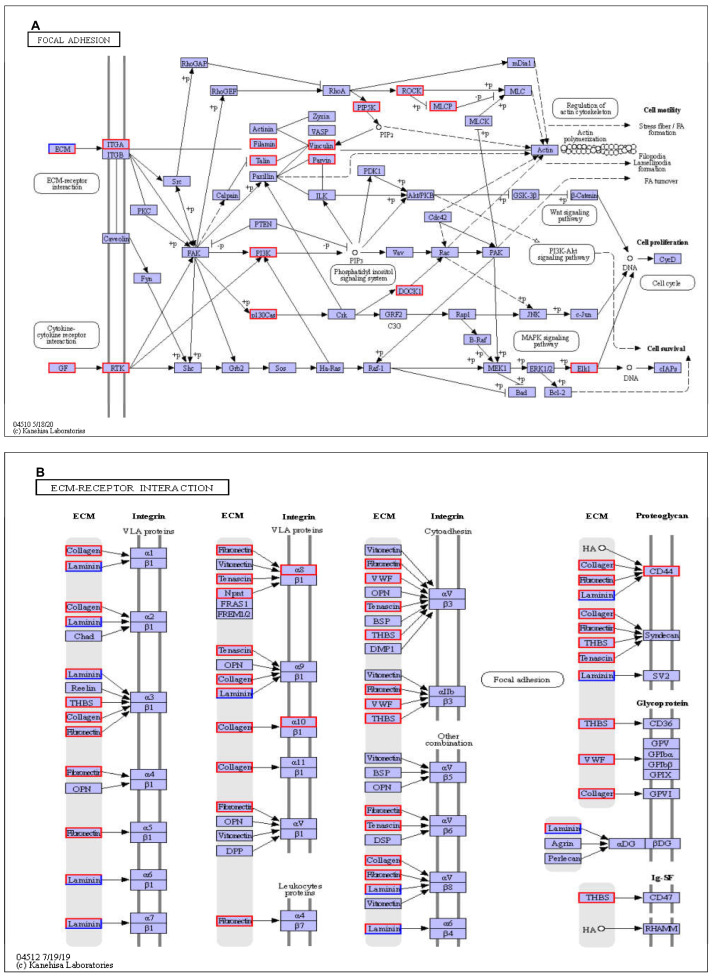
Significantly altered KEGG pathways in LAPST vs. CPG group comparison. (**A**) Focal adhesion signaling pathway’s DEG expression patterns. (**B**) ECM–receptor interactions; DEG expression patterns. Red highlighted genes indicate patterns of upregulated expression.

Focal adhesions help cells attach to the extracellular matrix (ECM) by connecting them via the actin cytoskeleton [69,70]. Surface molecules regulate cell-extracellular matrix (ECM) interactions, impacting various cellular processes, including proliferation, adhesion, migration, and differentiation [69]. Pathogens usually disrupt these adhesion molecules to breach epithelial barriers. Thus, protecting these interactions is vital for maintaining cellular functions and safeguarding epithelial integrity [71]. The findings indicate that probiotic mixtures can preserve cell structures, functions, and epithelial integrity even during *Salmonella* infection, as seen by the 33 and 18 elevated DEGs in the focal adhesion pathway and ECM–receptor interaction (Figure 5A,B).

Similarly, 16, 11, 9, and 9 upregulated DEGs were found in the inositol phosphate metabolism, tryptophan metabolism, glycine, serine, and threonine metabolism, and arginine and proline metabolism in the LAPST group as compared to CPG (Figure S6A,B and Figure S7A,B), which aligns with previous findings by Wu et al. [72], Zhang et al. [73], and Lin et al. [74].

The gut is a dynamic metabolically active organ, with metabolic abnormalities potentially exacerbating various intestinal diseases [75]. We performed a non-targeted LC-MS analysis contrasting the LAPST and CPG groups to evaluate the impact of probiotics on fecal intestinal metabolites. A total of 1520 significantly altered metabolites, with 1215 upregulated and 305 downregulated (Figure 3A), were mapped onto KEGG metabolic pathways to investigate possible functional effects on various biochemical and physiological mechanisms. These pathways include the arginine and proline metabolism, pantothenate and CoA biosynthesis, bile secretion, D-amino acid metabolism, propanoate metabolism, aminoacyl-tRNA biosynthesis, primary bile acid biosynthesis, amino acid biosynthesis, protein digestion and absorption, valine, leucine, and isoleucine biosynthesis, taurine and hypo-taurine metabolism, and phenylalanine metabolism (Figure 3C and Figure S8). Valine, leucine, and isoleucine biosynthesis are essential for protein synthesis and energy metabolism, which are critical to the host’s response to *Salmonella* infection [76]. Parallel studies were conducted by Shao et al. [77].

Amino acids are essential in biological functioning, acting as energy sources and building blocks for several biosynthetic processes in the body [78]. Gang Wang et al. showed that *L. reuteri* upregulated the amino acid metabolism [78], which is consistent with our study, where amino acid metabolism pathways were upregulated by probiotic supplementation. As the host negotiates nutritional competition and immunological responses, amino acid biosynthesis and aminoacyl-tRNA production become critical [79]. The proline and arginine metabolism regulates nitric oxide synthesis and boosts immunological responses, improving host defense systems and overall cellular function [80,81]. The process by which dietary proteins are broken down into amino acids and then absorbed in the small intestine mostly depends on protein digestion and absorption [82]. In line with our results, Jiajun Yang et al. demonstrated that probiotic *L. salivarius* increased arginine and proline production, consequently enhancing pigs’ nutritional digestion, absorption, and immunity [71]. Similarly, the taurine metabolism may regulate oxidative damage and inflammation in intestinal infections [83]. In our study, 3, 9, 5, 7, and 3 DEM pathways were found in valine, leucine, and isoleucine biosynthesis, the protein digestion and absorption pathway, the arginine and proline metabolism, amino acid biosynthesis, and the taurine and hypo-taurine metabolism, respectively (Figure S9A–C and Figure S10A,B). These metabolic pathways play crucial roles in boosting immunity, maintaining gut barrier integrity, and fine-tuning essential metabolic processes in the context of *Salmonella* infections.

## 5. Conclusions

In conclusion, our findings strongly support the efficacy of a well-designed combination of *L. acidophilus* and *L. plantarum* in suppressing *Salmonella* infection in mice. Our research investigated how probiotics affect gene expression and metabolite profiles post-infection, showing a significant increase in immunity, barrier integrity, and metabolic pathways in the probiotic-treated group compared to the untreated group. The activation of MAPK, PI3K/Akt, AMPK, and FoxO signaling pathways signifies a robust defense mechanism against *Salmonella*. Moreover, probiotics maintained their cellular functions and epithelial integrity, with increased DEGs in focal adhesion and ECM–receptor interaction pathways. A metabolomic analysis revealed changes in critical defense and cellular function pathways, showcasing the potential of probiotics in mitigating *Salmonella* adverse effects. These results emphasize the significance of probiotics in modulating host responses and effectively combating *Salmonella* infections.

## 6. Limitations

It is worth highlighting that our study utilized a combination of *L. acidophilus* and plantarum strains without examining their individual effects. Additionally, the study was conducted exclusively on mice; therefore, caution should be exercised when extrapolating the findings to humans. Subsequent research could focus on analyzing these strains separately to assess potential variations in effectiveness. Moreover, it is essential to conduct additional studies to clarify possible differences in effects and identify the best combinations for the most beneficial symbiotic or synergistic benefits. This manuscript is a foundation for future human investigations to evaluate the effects. The search for enhanced probiotic formulations presents a new frontier in our understanding of microbial therapies, necessitating continual investigation and refinement in the changing landscape of probiotics research.

## Data Availability

The study’s datasets, including metadata and raw/processed data, are available in online repositories via the assigned accession number at NCBI (PRJNA1056116).

## Supplementary Materials

The following supporting information can be downloaded at: https://www.mdpi.com/article/10.3390/genes15040435/s1. Figures S1–S10; Tables S1–S8.
