# Supplementary material for: Novel Synergistic Probiotic Intervention: Transcriptomic and Metabolomic Analysis Reveals Ameliorative Effects on Immunity, Gut Barrier, and Metabolism of Mice during Salmonella typhimurium Infection
Source: Genes (Basel). 2024 Mar 29;15(4):435. doi: 10.3390/genes15040435 (PMC11050207; doi:10.3390/genes15040435)
Supplement: Supplementary file 1 [file genes-15-00435-s001.zip › Supplementary.pdf]

genes

## Supplementary

*Article*

# Novel Synergistic Probiotic Intervention: Transcriptomic and Metabolomic Analysis Reveals Ameliorative Effects on Immunity, Gut Barrier, and Metabolism of mice during *Salmonella Typhimurium* Infection

**Muhammad Junaid1, Hongyu lu1, Yixiang Li1, Yu Liu1, Kefei liu2, Ahmad Ud Din3, Zhong Quan Qi1*, Jianhua Yan1***

1. Medical College, Guangxi University, Nanning 530004, Guangxi, China
2. Plants for Human Health Institute, Department of Food Bioprocessing and Nutrition Sciences North Carolina State University 600 Laureate Way, Kannapolis, NC 28081, USA

*Correspondence: J.Y (jianhuayan@gxu.edu.cn: Z.Q (yxyyz@gxu.edu.cn))

**Table Supplementary 1**. Primers used for Validation of RNA Sequencing by qRT-PCR.

| Gene | Primers |
|---|---|
| β-actin | F-AGAGAAGCTGTGCTATGTTGCT |
| | R-GGAACCGCTCGTTGCCAATA |
| SOD-1 | F-GCGGATGAAGAGAGGCATGT |
| | R-TTCCACCTTTGCCCAAGTCA |
| IL-10 | F-GCAAGGGTGTCTCCTTCCTC |
| | R-CTTGTTACACTCGCCCCCTT |
| Cldn-1 | F-GGCTTCTCTGGGATGGATCG |
| | R-TTTGCGAAACGCAGGACATC |
| Cox2 | F-TCAAGCCTAGCCCCAACACC |
| | R-TGCTGTGGTGAGCCCATGTA |
| Tnfsf14 | F-GATCGGTCCCCAAAGGGATG |
| | R-CCACTTGGTGGTTTGTGAGTG |

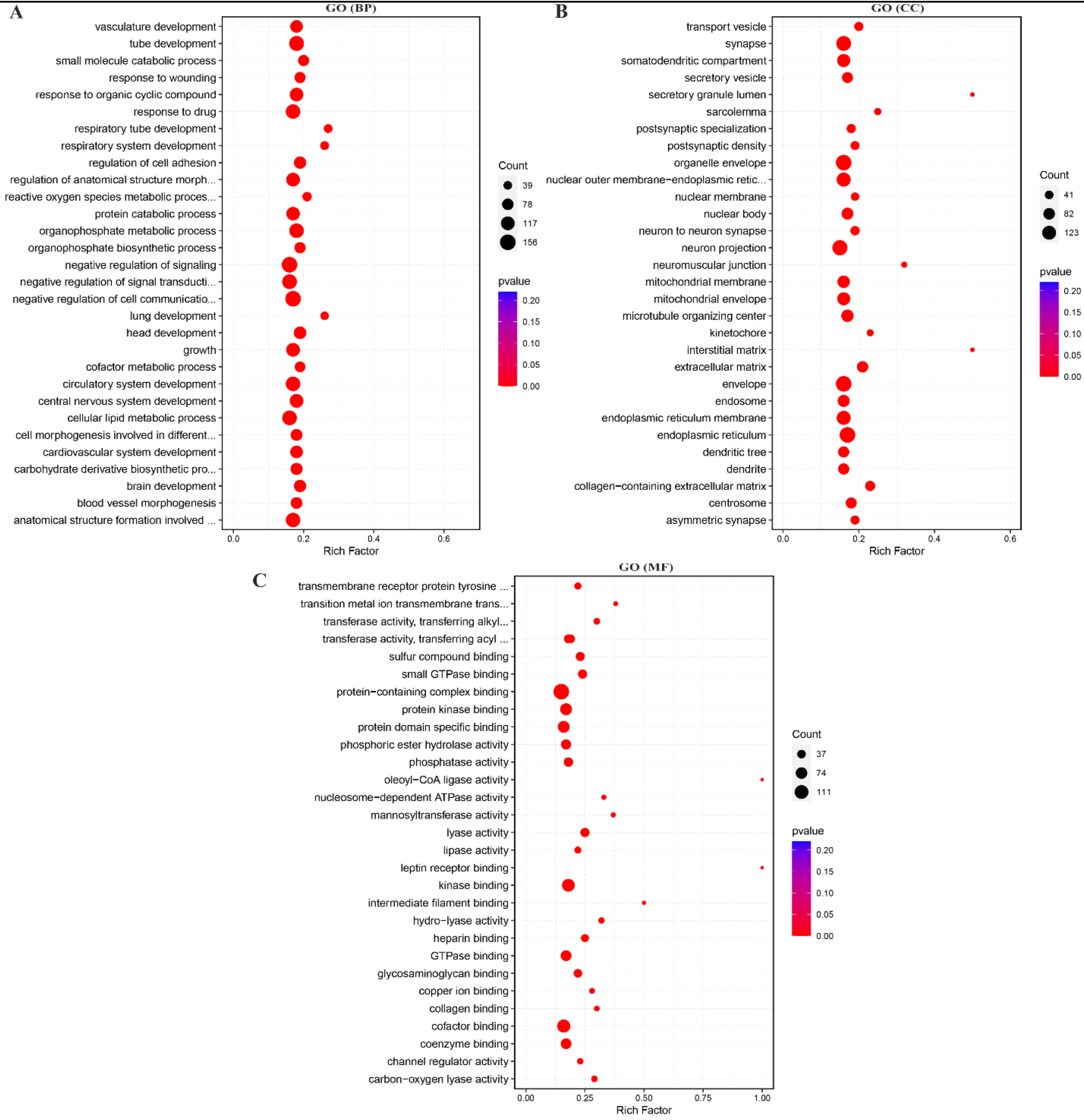


Figure Supplementary 1. Gene ontology (GO) functional enrichment analysis of up and downregulated DEGs between LPST vs CPG group. (A) Biological process. (B) Cellular component. (C) Molecular Function. The size of the bubble represents the number of genes in the set. The P value is indicated by the color of the bubble.

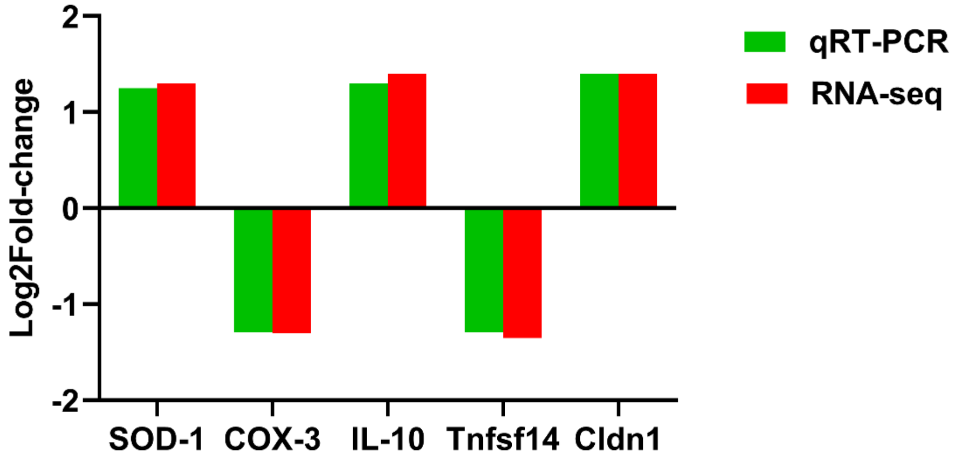


**Figure Supplementary 2.** DEGs validation by qRT-PCR

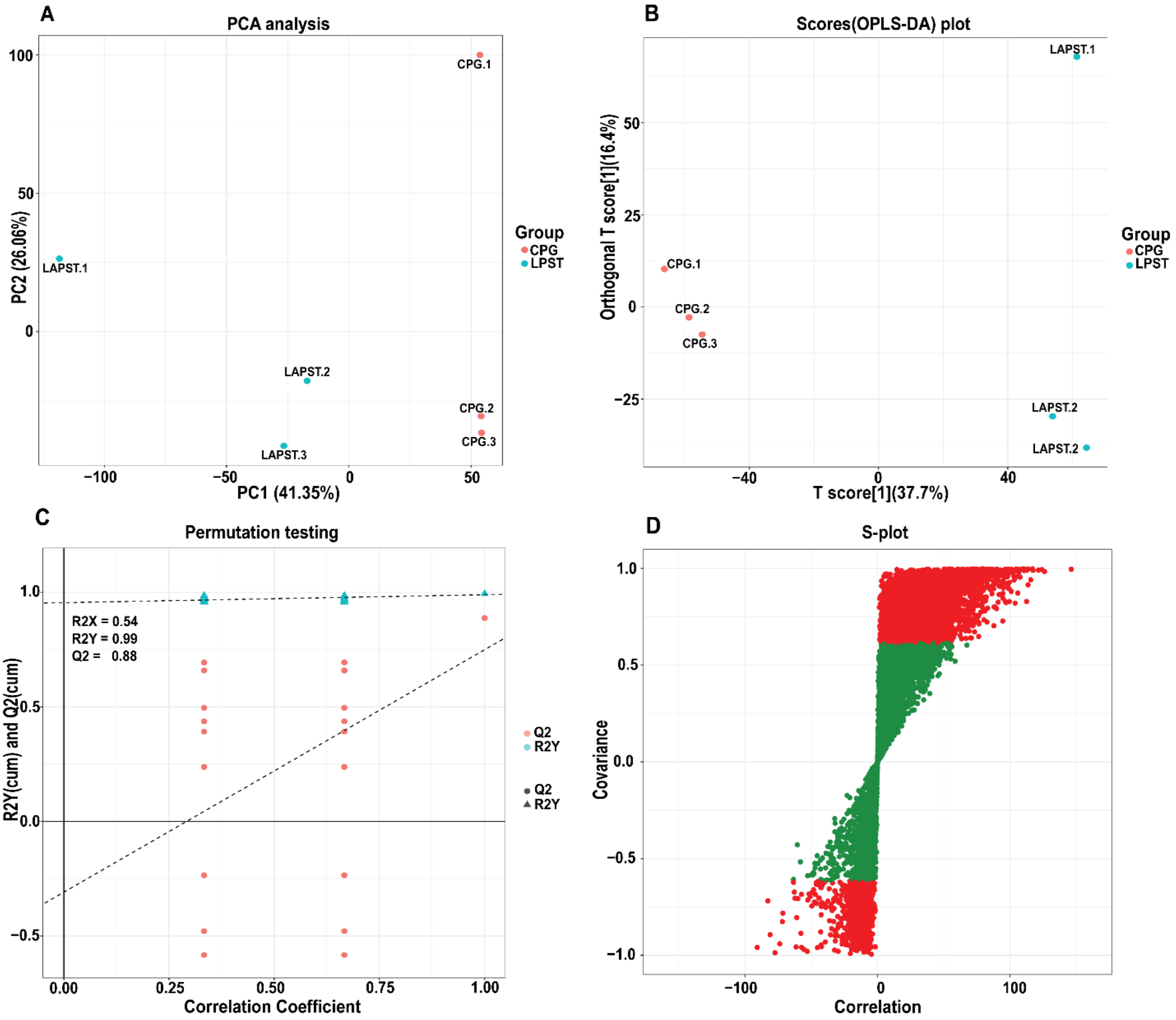


**Figure Supplementary 3.** Orthogonal partial least squares Discriminant analysis (OLPS-DA) model. (A) PCA analysis showed significant difference between groups. (B) OPLS-DA model. (C) Permutation test. (D) S-plot.

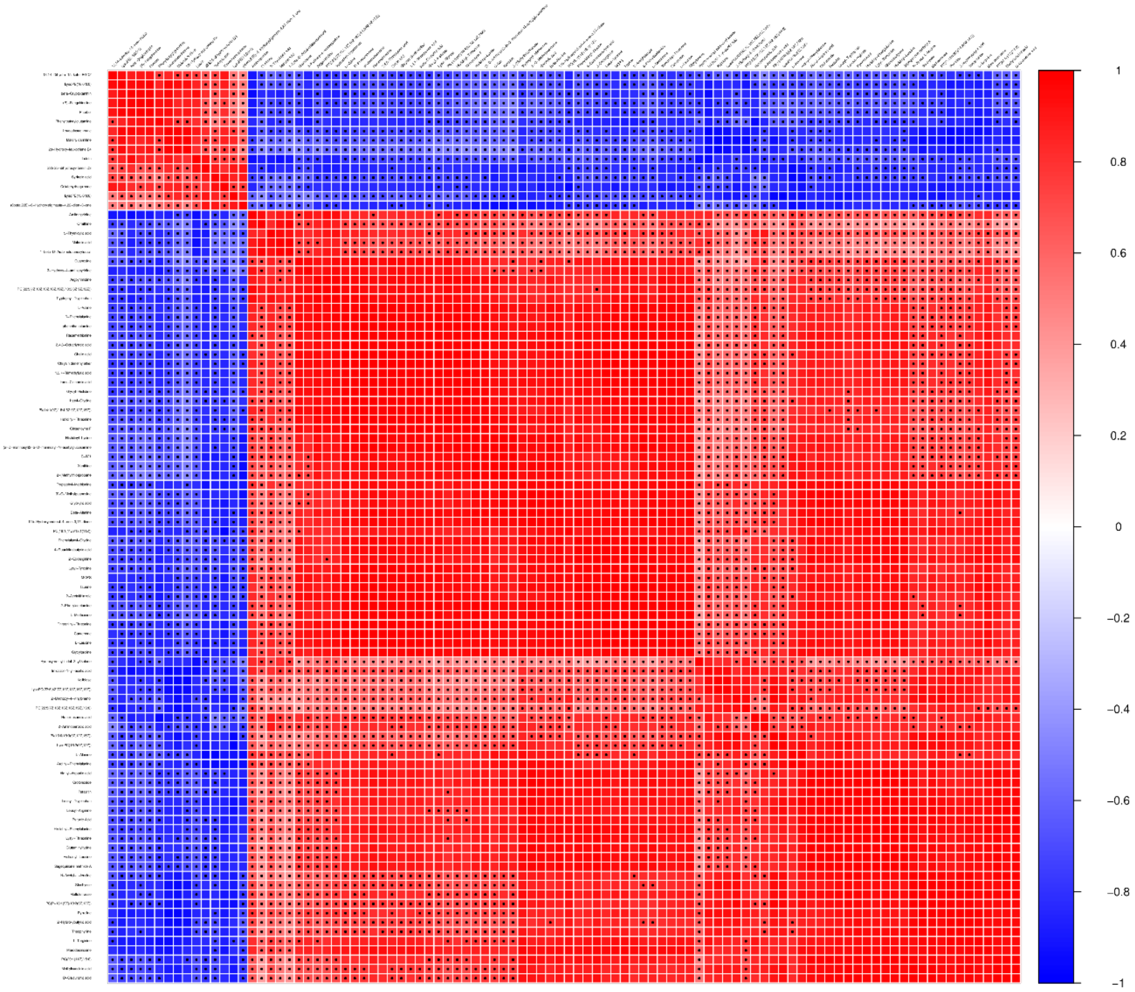


**Figure Supplementary 4.** LC/MS visual Correlation Analysis of Top 100 metabolites between LAPST vs. CPG.

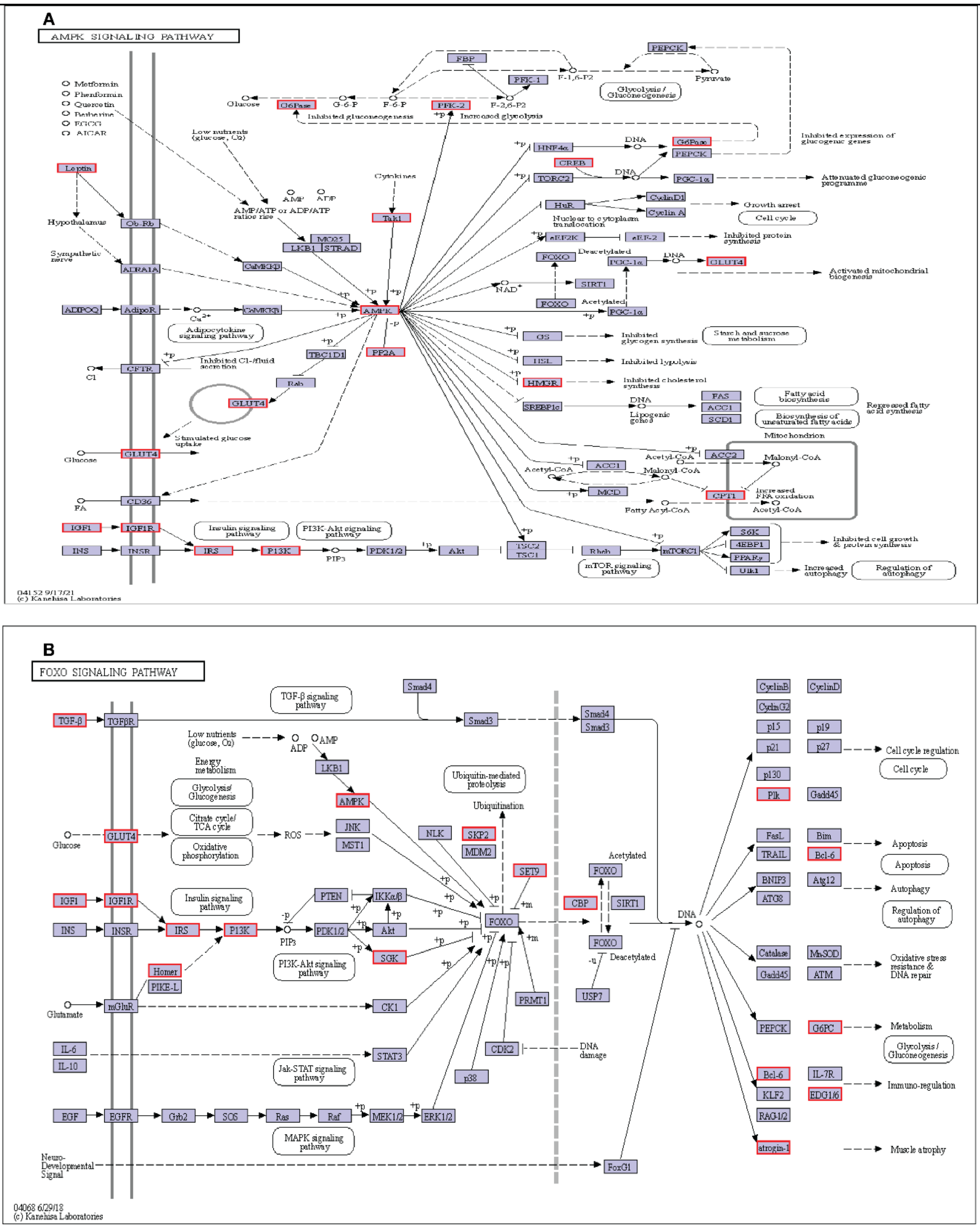


**Figure Supplementary 5**. Significantly altered KEGG pathways in LAPST versus CPG group comparison. (A) AMPK signaling pathway's DEG expression patterns. (B) Foxo signaling pathway's DEG expression patterns. Red-highlighted genes indicate patterns of up-regulated expression.

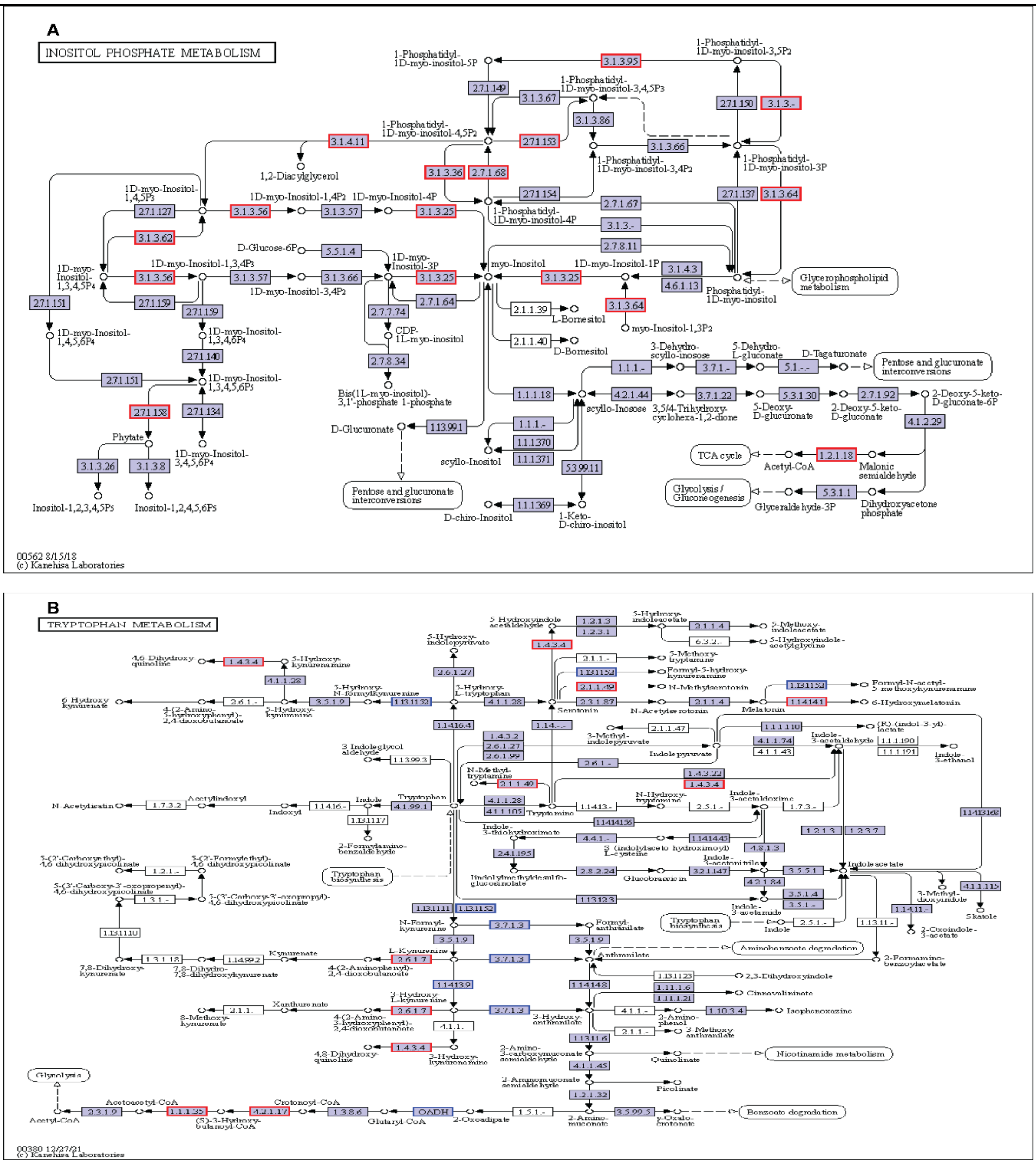


**Figure Supplementary 6**. Significantly altered KEGG pathways in LAPST versus CPG group comparison. (A) Inositol phosphate metabolism DEG's expression patterns. (B) Tryptophan metabolism DEG's expression patterns. Red-highlighted genes indicate patterns of up-regulated expression.

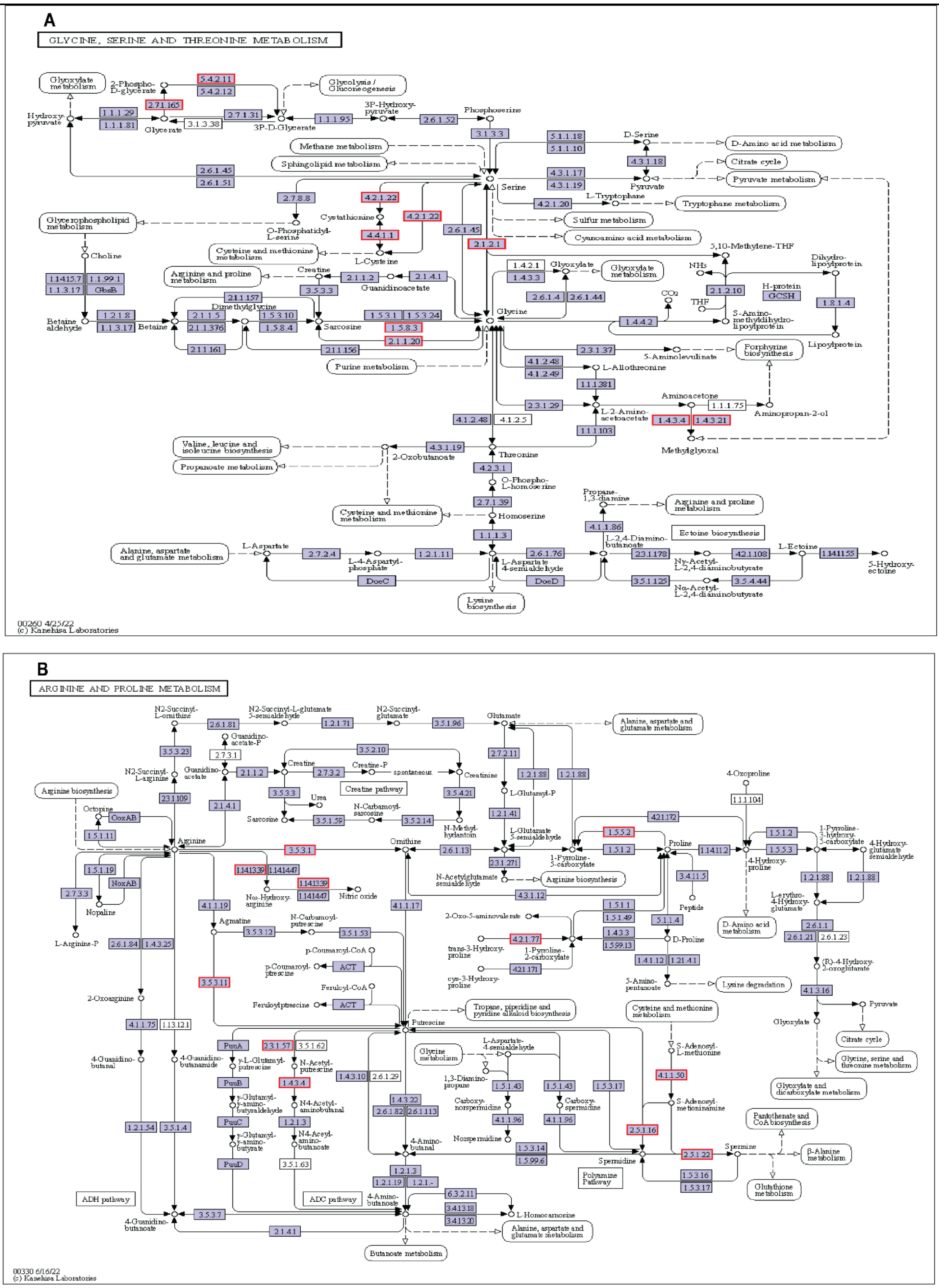


**Figure Supplementary 7.** Significantly altered KEGG pathways in LAPST versus CPG group comparison. (A) Glycine, serine and threonine metabolism DEG's expression patterns. (B) Arginine and proline metabolism DEG's expression patterns. Red-highlighted genes indicate patterns of up-regulated expression.

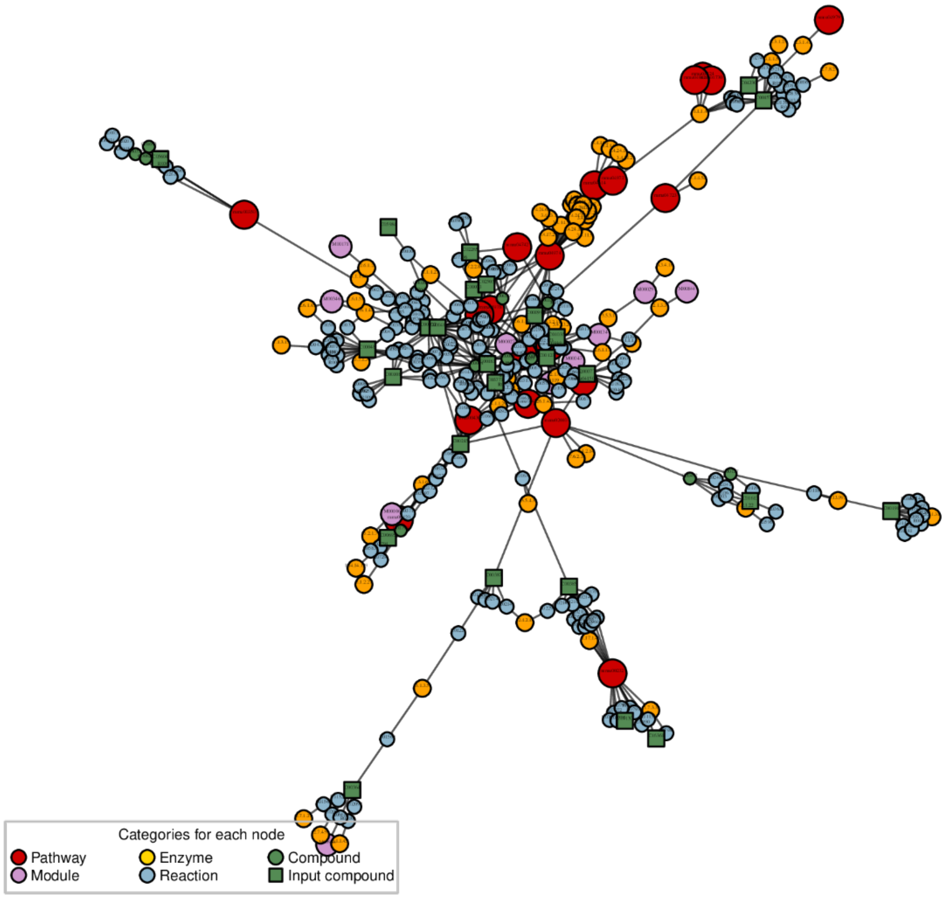


48

**Figure Supplementary 8.** Network analysis showed enriched KEGG pathways between LAPST versus CPG group. Red nodes indicate enriched pathways. 49 50

51

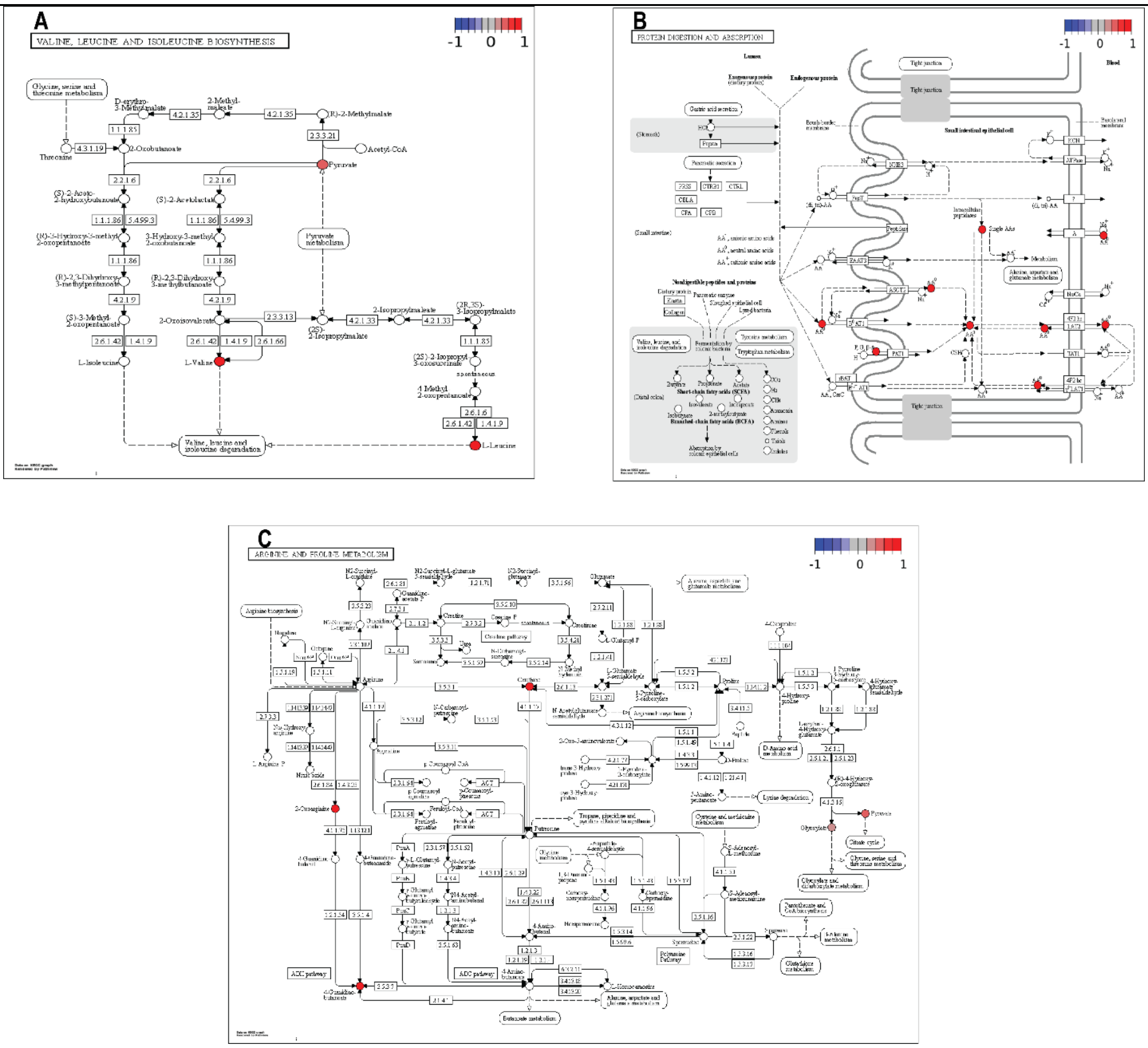


**Figure Supplementary 9.** Significantly altered KEGG metabolic pathways in LAPST versus CPG group comparison. (A) Valine, leucine and isoleucine biosynthesis pathways DEM's expression patterns. (B) Protein digestion and absorption pathway DEM's expression patterns. (C) Arginine and proline metabolism pathways DEM's expression patterns. Red-highlighted genes indicate patterns of up-regulated expression.

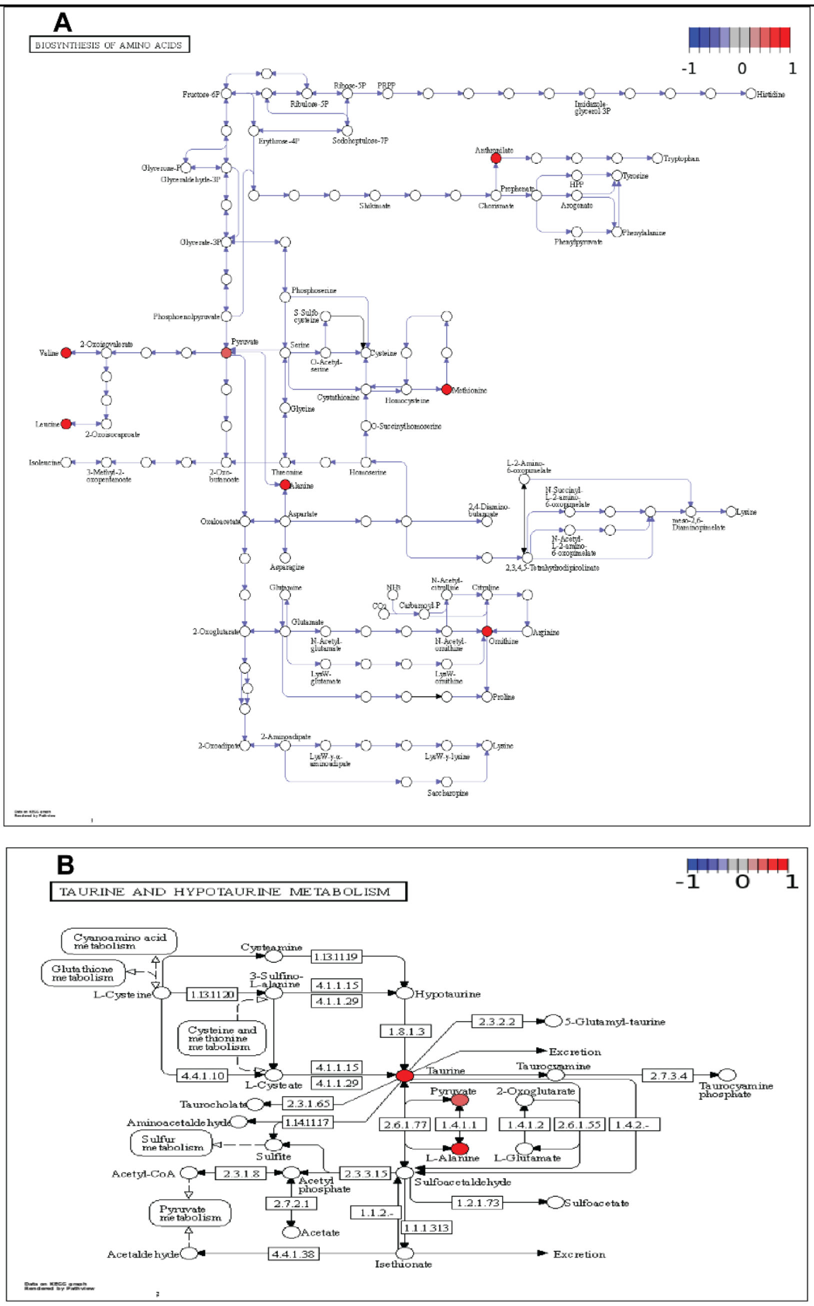


**Figure Supplementary 10.** Significantly altered KEGG metabolic pathways in LAPST versus CPG group comparison. (A) Amino acid biosynthesis pathways DEM's expression patterns. (B) Protein digestion and absorption pathway DEM's expression patterns. (C) Taurine and hypotaurine metabolism pathways DEM's expression patterns. Red-highlighted genes indicate patterns of up-regulated expression.
